# Icaritin and lenvatinib treatment for unresectable localized progressive pancreatic cancer: a report of six cases

**DOI:** 10.1080/07853890.2025.2512436

**Published:** 2025-06-05

**Authors:** Xiaolong Liu, Jianguo Lu, Xinyu Dong, Yizhuo Zhang, Guixing Jiang, Yanlong Cao, Defei Hong

**Affiliations:** a Department of General Surgery, Sir Run Run Shaw Hospital & Zhejiang University School of Medicine, Hangzhou, China; b Department of General Surgery, Tangdu Hospital & Fourth Military Medical University, Xi’an, China

**Keywords:** Icaritin, combination therapy, pancreatic cancer, lenvatinib, advanced treatment strategies

## Abstract

**Background:**

Pancreatic cancer is very difficult to detect in its early stages, and its rapid progression and poor prognosis make it a significant therapeutic challenge. Existing treatment modalities, whether standalone or combined, offer limited efficacy, highlighting an urgent need for more effective and less toxic therapeutic strategies.

**Patients:**

We analyze six cases of pancreatic cancer, each undergoing a novel treatment regimen integrating chemotherapy, targeted therapy, immune therapy, and icaritin.

**Results:**

The outcomes demonstrate varying degrees of improvement in all cases. Partial responses were observed in cases 1 and 3, with some tumor shrinkage. Cases 4 and 5 showed limited relief following a slight decrease in tumor size. In cases 2 and 6, no significant progression of the lesion was observed. These results highlight the potential efficacy of this multifaceted approach.

**Conclusion:**

Integrating icaritin with chemotherapy, immunotherapy, and/or targeted therapies shows potential as an exploratory approach in managing advanced, locally progressive, or metastatic pancreatic cancer, though further validation is required.

## 1. Introduction

Pancreatic cancer progresses swiftly and has a poor prognosis, making it a leading cause of mortality among malignant tumors. In the United States in 2022, it ranked as the tenth most prevalent cancer, with 62,210 new cases [1]. Although it is less common than some other cancer types, its high malignancy contributes to it being the third leading cause of cancer-related deaths [2,3]. Standard treatments such as surgery, radiotherapy, and chemotherapy achieve only a 43.9% 5-year survival rate for patients with localized disease in the United States, and patients with inoperable localized or metastatic pancreatic cancer face drastically lower survival rates of 14.7% and 3.1%, respectively [2]. In China, where more than 70% of cases are diagnosed at advanced or metastatic stages, the 5-year survival rate is 7.2%, the lowest among all cancers [4]. The majority of patients present with advanced disease, and only a minority of 15–20% are candidates for surgical intervention [5].

The use of gemcitabine monotherapy, which was initiated by Burris, has become a cornerstone in the treatment of progressive pancreatic adenocarcinoma [6]. Research has also validated the efficacy of various drug combinations, although these are associated with increased rates of side effects [7–9]. It has been suggested that most patients will not benefit from surgery alone in the early stage of pancreatic cancer after initial diagnosis, owing to the presence of micrometastases that are undetectable in radiological examinations before surgery, raising the question of whether chemotherapy, including neoadjuvant treatment and adjuvant treatment, should be carried out. Indeed, a study has proved that neoadjuvant chemoradiation can have survival benefits in such cases [10]. As many patients cannot tolerate the adverse effects of the regimen consisting of folinic acid, fluorouracil, irinotecan hydrochloride, and oxaliplatin (also called FOLFIRINOX) regime in China, other regimens such as albumin paclitaxel and gemcitabine (AG) or gemcitabine and S-1 (GS), have been considered as alternatives [8]. Previous studies have confirmed that AG and S-1 have synergistic effects [11,12]. Nowadays, the AG regimen represents one of the standard regimens for first-line treatment of metastatic pancreatic adenocarcinoma (mPDAC), even though it is considered the best option in Asia [13,14], however, the significant toxicity of these combination therapies often limits their clinical applications, including anemia, thrombocytopenia, and leukopenia caused by bone marrow suppression. Thus, there is an urgent need for treatment options that are both more effective and less toxic than current regimens for pancreatic carcinoma, especially for advanced pancreatic cancer.

Icaritin is derived from the deglycosylation of icariin, the primary active constituent of epimedium extract [15,16]. As an innovative small-molecule immunomodulator with proprietary intellectual property, icaritin represents a global first-in-class drug. Many pharmacological studies have suggested the efficacy of icaritin in suppressing tumor cell growth in breast, lung, liver, and prostate cancers [17]. Numerous studies have shown that icaritin has the property of inhibiting cancer progression and confirmed that it can inhibit tumor cell growth by controlling multiple signaling pathways, including obstructing the cell cycle, restraining cell proliferation, invasion, and migration, regulating autophagy, and inducing cell apoptosis and differentiation [18,19]. Further studies have demonstrated a connection between the anticancer activity of icaritin and its metabolites and certain transcription factors (NF-κB, STAT3, β-catenin, etc.), growth factors (TGF-β, VEGF, EGF, etc.), and protein kinases (AKT, MAPK, PI3K, JNK, etc.) [20–22]. The targeted therapeutic effects of icaritin could thus be expected to enhance clinical therapeutic efficacy. Preclinical *in vivo* pharmacodynamic studies have shown that epimedin has significant tumor growth inhibitory effects across various models. As an immunomodulator, icaritin inhibits inflammatory signaling pathways, reduces inflammatory factor release, and improves antigen presentation. As an emerging candidate drug for anticancer treatment in the clinical application, icaritin is currently approved as a first-line treatment for hepatocellular carcinoma, in which it promotes apoptosis and inhibits hepatocyte proliferation by downregulating AFP gene expression, enhancing its anti-tumor efficacy [23,24]. In a past case study, icaritin and lenvatinib were applied for the treatment of locally advanced unresectable pancreatic cancer and achieved the effect of phase reduction and partial remission [25].

In the present study, based on our experience and related research, we evaluated six patients with advanced, locally progressive, or metastatic pancreatic cancer who underwent a treatment regimen incorporating chemotherapy, immunotherapy, targeted therapy, and icaritin, either post-surgery or as an alternative to surgery. Following icaritin and associated with chemotherapy and the targeted drugs, the preliminary results of this study indicate that all patients exhibited varying degrees of tumor control and remission, and tolerated adverse reactions. More evidence-based research is still needed to further confirm.

## 2. Patients

### 2.1. Ethical approval statement

This study was approved by the Ethics Committee of Sir Run Run Shaw Hospital, Zhejiang University School of Medicine (2024-2008-01) and was conducted in accordance with the Declaration of Helsinki. The study was conducted in accordance with the local legislation and institutional requirements. We confirm that written consent to publish these details has been obtained from all individuals (or their legal guardians).

### 2.2. Case 1

**Figure 1.**
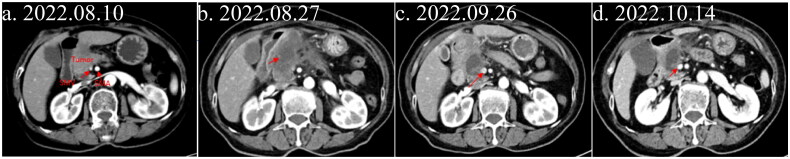
Abdominal imaging changes in pancreatic cancer during treatment with lenvatinib and icaritin. The red arrow indicates the tumor site. (a) Enhanced abdominal CT at admission. (b) Enhanced abdominal CT after initial treatment. (c) Enhanced abdominal CT at 40-day follow-up. (d) Enhanced abdominal CT at 2-month follow-up.

A 79-year-old female patient was admitted to the hospital presenting with a 1-week history of upper mid-abdominal distension and pain, accompanied by the discovery of a pancreatic lesion 6 days prior. The patient’s pain score was 1 point on a scale of 1 to 10. Laboratory investigation revealed elevated blood glucose (11.4 mmol/L) and tumor marker, Carbohydrate antigen (CA) 19-9 (at 69.8 IU/mL); these, combined with abdominal imaging results, led to a preliminary diagnosis of pancreatic head cancer. Treatment with lenvatinib and icaritin regimen (lenvatinib 8 mg orally once daily + icaritin 3 capsules (300 mg) orally twice daily) commenced on August 15, 2022. After 40 days, imaging showed a decrease in the pancreatic head-neck mass from 57 × 31 mm to 35 × 28 mm. On October 14, 2022, a month after treatment, a computed tomography (CT) scan indicated further reduction to approximately 31 × 25 mm, with mild enhancement at the edges and reduced stenosis of the superior mesenteric vein (SMV) (Figures 1a-1d). This represented a 45.6% tumor shrinkage, classified as partial response (PR), and improved SMV stenosis. The tumor stage was downgraded from pre-treatment venous unresectable locally progressive (T3N0M0, stage IIA) to post-treatment resectable (T2N0M0, stage IB). Surgery was advised by the multidisciplinary team but declined by the patient and her family. Treatment cessation followed owing to financial constraints. Subsequent abdominal CTs showed tumor progression and increased size (Supplementary Figure 1), with ferritin levels rising to 799.0 μg/L and CA199 from 30.52 U/mL to 501.6 U/mL. The patient’s overall survival was 8 months.

### 2.3. Case 2

**Figure 2.**
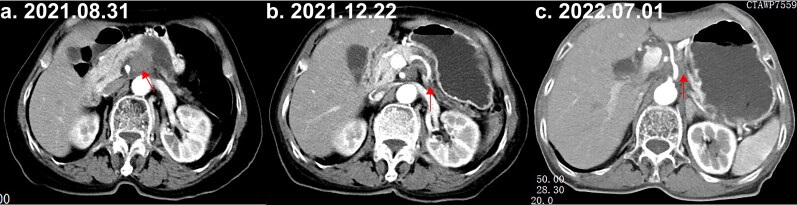
Abdominal CT showing pancreatic cancer post-chemotherapy and immunotherapy. The red arrow highlights the tumor locations. (a) Initial abdominal CT scan at admission. (b) Abdominal CT scan after AG and camrelizumab treatment. (c) Abdominal CT scan following completion of the second chemotherapy course with FOLFIRINOX.

A 75-year-old woman with controlled hypertension was admitted for left upper abdominal distension and pain persisting for 2 months. Her physical examination was largely unremarkable; and her laboratory work-up revealed a slight elevation in blood glucose (7.32 mmol/L) and CA19-9 levels (305.90 IU/mL). Initial CT imaging suggested pancreatic body cancer with potential celiac trunk and superior mesenteric artery involvement (Figure 2a). The patient began chemotherapy on September 1, 2021, receiving four cycles of AG and camrelizumab. This treatment resulted in a 28.6% lesion reduction, with the size of the tumor decreased from 27 × 28mm to 20 × 14mm, classified as stable disease (SD) by December 1, 2021 (Figure 2b). She then underwent six cycles of the FOLFIRINOX regimen from December 27, 2021, to May 24, 2022. However, imaging on July 1, 2022, showed a 50% increase in tumor size, indicating progressive disease (PD) (Figure 2c). The patient subsequently started treatment with a GS regimen combined with icaritin (6 capsules (2.4 g) orally twice daily) from July 1, 2022, to February 9, 2023. During this period, imaging results indicated SD with no significant changes, which reveals that there is a 3 cm mass on the dorsal side of the pancreatic body without any changes, and the imaging examination no longer shows liver metastasis (Supplementary Figure 2). Unfortunately, disease progression followed treatment discontinuation, leading to the patient’s demise after an overall survival of approximately 18 months.

### 2.4. Case 3

A 77-year-old female patient was admitted to our hospital due to a pancreatic lesion. She had a history of hypertension, which was well-controlled with medication. Initial tests indicated slightly elevated blood glucose (7.32 mmol/L) and tumor markers (CA19-9: 20.96 IU/mL, CA15-3: 16.30 U/mL, CA125: 48.20 U/mL). Admission CT suggested a malignant pancreatic tail tumor with suspected metastasis (Supplementary Figure 3). On May 16, 2022, the patient underwent laparoscopic distal pancreatectomy, total splenectomy, partial colectomy, and intestinal adhesion. The postoperative pathology report identified a moderate-to-low differentiated adenocarcinoma measuring 6.8 × 5.54 cm, with clear margins and no lymph node involvement (0/15). It was classified as pT3N0Mx according to the 8th edition of the American Joint Committee on Cancer (AJCC) staging system. There was no involvement of the colon, spleen, or adrenal tissues. Her postoperative treatment, starting on June 18, 2022, included one cycle of AG regimen chemotherapy, after which she declined further chemotherapy. Imaging on February 13, 2023 revealed intrahepatic metastasis, then the patient recieved six cycles of AG regimen + camrelizumab therapy (Supplementary Figure 4). Following tumor progression, the patient received 2 months of icaritin (icaritin 6 capsules (2.4 g) orally twice daily) treatment. Subsequent follow-up imaging (Supplementary Figure 5 and Supplementary Table 1) on May 24, 2023, showed a reduction in liver metastases and retroperitoneal lymph nodes, which liver metastasis reduced from 37 × 26mm to 16 × 10mm, and retroperitoneal lymph nodes declined to 8 mm from 11 mm after 3 months treatment, indicating persistent PR. It is noteworthy that the patient is still alive.

### 2.5. Case 4

**Figure 3.**
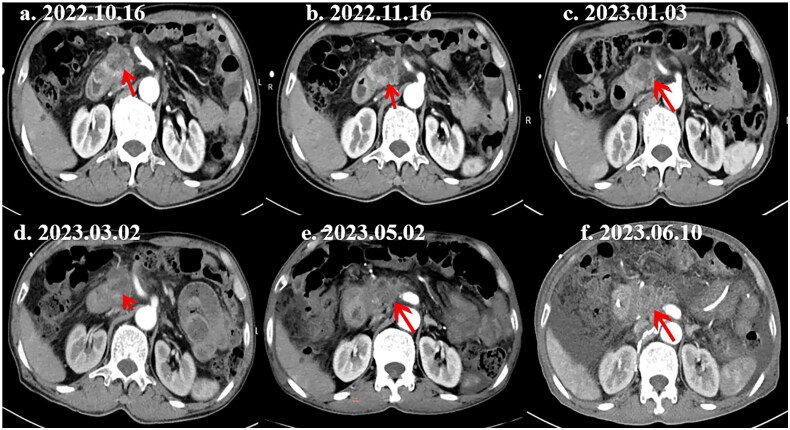
Abdominal CT imaging after treatment with lenvatinib and icaritin (red arrow: tumor sites). (a) Pre-treatment: multicystic mass in pancreatic head. (b) Post-treatment: reduced mass size, changed enhancement patterns, and enlarged lymph nodes. (c–f) During and after icaritin treatment: tumor lesions stable at 2.5–3 cm, later increasing to 3.5–4 cm.

A 74-year-old male was admitted to our hospital for one month of abnormal liver function. Mild jaundice was observed in his skin and sclera, and no enlargement of superficial lymph nodes was detected. Biochemical tests showed elevated liver transaminases and bilirubin levels, and serum CA19-9 (257.8 IU/mL) and CA125 (36.8 U/mL) levels. Admission CT scans indicated a likely tumor in the pancreatic head (Supplementary Figure 6). An ultrasonographic endoscopic biopsy with histology on October 18, 2022, revealed glandular epithelial disruption and cellular anomalies in the blood clot from the pancreatic puncture, consistent with adenocarcinoma. Immunohistochemistry results were S100P/control (+), Muc-1 (+), and CD10 (some weak +). The patient opted for conservative treatment after declining surgery, as recommended by the multidisciplinary team. He chose lenvatinib and icaritin regimen (lenvatinib 8 mg orally once daily + icaritin 6 capsules (2.4 g) orally twice daily). Imaging after 1 month showed a slight reduction in tumor mass, with the size of the tumor decreased from 34 × 31mm to 31 × 28mm, representing a reduction of approximately 17.6% (Figure 3). The treatment, spanning from October 24, 2022, to May 8, 2023, consistently demonstrated tumor stability without progression, as evidenced by imaging on January 3, March 2, May 2, and June 10, 2023. The patient is currently alive.

### 2.6. Case 5

A 57-year-old woman presented with left upper abdominal pain lasting for 3 months. Upon admission, she exhibited mild jaundice of the skin and sclera, without any other significant abnormalities. Hematological tests revealed elevated levels of hepatic aminotransferases and bilirubin and serum CA19-9 (49.66 IU/mL). Admission CT scans suggested pancreatic cancer invading the splenic artery (Supplementary Figure 7). The patient underwent laparoscopic radical antegrade modular pancreatosplenectomy (RAMPS) on November 11, 2021. Post-operative pathology revealed a 3.5 × 2 cm moderately differentiated adenocarcinoma with ependymal layer infiltration, nerve involvement, intraparenchymal embolus, and cancer metastasis in one of seven lymph nodes. The tumor was staged as pT2N1Mx (AJCC 8th edition). The patient received a postoperative AG regimen + camrelizumab treatment from January 26, 2022, to August 15, 2022. Subsequent imaging revealed tumor progression (Supplementary Figure 8). She declined further recommended treatment on October 28, 2022, and continued to take lenvatinib and icaritin regimen (lenvatinib 8 mg orally once daily + icaritin 6 capsules (2.4 g) orally twice daily) for 6 months (October 28, 2022, to April 2023), which resulted in substantial resolution of pre-existing liver metastases. However, after discontinuing treatment, tumor progression was observed and the patient passed away on April 16, 2023, with an overall survival of 17 months.

### 2.7. Case 6

A 68-year-old woman was admitted following a colonoscopy that had led to the diagnosis of a malignant rectal lesion 2 weeks before. The colonoscopy revealed lesions in the rectum and distal transverse colon, with the malignancy confirmed by biopsy. Abdominal imaging showed a suspicious lesion in the pancreatic tail, suggestive of malignancy (Supplementary Figure 9a). Tumor marker tests on August 20, 2022, indicated elevated CA19-9 (3009.0 IU/mL) and alpha fetoprotein (AFP; 14.60 ng/mL) levels. Pancreatic biopsy pathology confirmed moderate-to-low differentiated adenocarcinomas within fibrous connective tissue. The patient started the GP chemotherapy regimen (gemcitabine 1000 mg/m^2^ and cisplatin 25 mg/m^2^). However, the results of positron emission tomography (PET)-CT imaging on November 22, 2022, showed metastatic progression. Subsequently, on December 4, 2022, a GP regimen combined with icaritin (six capsules (2.4 g) orally, twice daily) was initiated. On December 30, 2022, CA19-9 levels had decreased to 181.0 IU/mL, and AFP had decreased to 12.40 ng/mL. The patient continued with oral icaritin, and a follow-up PET-CT examination on March 16, 2023, indicated SD. Then, radiation therapy (50 Gy/25 fractions) was administered on April 4, 2023. On May 23, 2023, CA19-9 and AFP levels were 5.86 IU/mL and 13.70 ng/mL, respectively, suggesting tumor improvement. Intensive CT imaging on May 24, 2023 (3.0 cm × 2.5 cm), confirmed a reduction in tumor lesions compared to August 18, 2022, with the tumor size decreasing from 4.3 cm to 3.0 cm. No new abnormalities were detected. The imaging also showed a reduction in the enlarged lymph nodes at the right diaphragmatic angle, a slight decrease in the extent of pancreatic tail lesions, and absorption of pelvic fluid compared to previous findings (Supplementary Figure 9b). The patient remains alive.

## 3. Discussion

Icaritin, which has been identified as a key flavonoid glycoside in epimedium extract [24], demonstrates a spectrum of pharmacological actions. The research underscores its efficacy in inhibiting tumor cell growth by targeting various molecular signaling pathways. Specifically, as described in the background, icaritin curtails tumor cell proliferation and migration, fosters cell differentiation and apoptosis, modulates autophagy, and disrupts the cell cycle [13]. Fundamental study has linked the anti-tumor properties of icaritin and its metabolites to the modulation of transcription factors (such as NF-κB, STAT3, β-catenin), protein kinases (including MAPK, AKT, JNK, PI3K), and growth factors (VEGF, TGF- β, EGF) [20–22,26]. For example, TGF-β is a major regulator of proteoglycan synthesis, and its deregulation is thought to be involved in the pathophysiological processes of pancreatic cancer. TGF-β has been reported to have a dual role in pancreatic cancer. A study has shown that rhoifolin—which, like icaritin, is an active flavonoid component—can significantly inhibit the TGF-β2/SMAD2 signaling pathway in pancreatic cells, which may contribute to its inhibitory effects on cancer cell migration and invasion [27]. Furthermore, studies indicate that icaritin augments anti-tumor activity and potentiates the efficacy of radiotherapy [28]. Given this, case 6 received adjuvant radiation therapy at a certain dose after treatment with icaritin to enhance the therapeutic effect. So far, the basic research related to icaritin has confirmed its clear anti-tumor effects in various tumors and has shown encouraging outcomes in exploratory clinical studies of liver cancer treatment [16,18]. Therefore, people believe that, as an emerging candidate drug, icaritin may become a potentially effective cancer treatment drug.

Considering the side effects of chemotherapy, most patients currently prefer to choose targeted, immune, or combined traditional Chinese medicine with fewer side effects for anti-tumor treatment. Comprehensive analysis of various reasons such as the patient’s previous treatment and concerns about chemotherapy side effects, the efficacy of icaritin and lenvatinib or AG/GS regimen against liver cancer and other solid tumors, in this case study, based on the patient’s condition, treatment status, and willingness to use medication, especially after communicating with patients and their families about possible adverse reactions, all pancreatic cancer patients chose to use icaritin in combination with lenvatinib or AG/GS regimen. Through the study of the limited number of cases mentioned above, we initially observed the preliminary and local efficacy of icaritin and lenvatinib or AG/GS regimen in the treatment of pancreatic cancer and noticed that the effect is better in pancreatic cancer patients with elevated CA199. Simultaneously, due to the experimental and exploratory nature of the case study, we did not strictly divide the experimental and control groups according to standardized clinical trial, only wanted to explore the initial effect of carnitine combined with lenvatinib or AG/GS regimen in advanced pancreatic cancer and whether the patient can tolerate the therapy. Some patients have undergone various treatments including chemotherapy, with some receiving surgery; this was often augmented with immune or targeted therapies, notably including icaritin. Despite the different durations of icaritin treatment, temporary partial tumor remission was frequently observed in post-treatment imaging, indicating the need for further exploration of its clinical effectiveness [29]. Such as liver metastasis decreased from 37 × 26mm to 16 × 10mm in case 3 after treatment 3 months later and a reduction in the pancreatic head-neck mass from 57 × 31 mm to 35 × 28 mm in case 1 after treatment 40 days later with icaritin alone and icaritin combined with lenvatinib. The related treatment is listed in Table 1 with a comprehensive summary of each case, detailing gender, age, symptoms, diagnosis, CA19-9 levels, treatment protocols, and outcomes. This table will be instrumental in elucidating the varied responses to and efficacy of icaritin alone and in combination with lenvatinib or chemotherapy across different cases. Using the data presented in Table 1, explore the potential role of these treatments in these scenarios and assess their suitability as a complementary treatment to lenvatinib or chemotherapy in pancreatic cancer.

**Table 1.** Summary of clinical characteristics, treatment approaches, and preliminary outcomes in pancreatic cancer cases.

Case	Gender	Age	Symptoms	Diagnosis	CA19-9 (IU/mL)	Treatment plan	Overall survival (outcome)
1	Female	79	Upper mid-abdominal distension and pain for 1 week, pancreatic mass identified for 6 days	Pancreatic head cancer	69.8	Lenvatinib + icaritin	8 months (PR, 45.6% tumor shrinkage)
2	Female	75	Left upper abdominal distension and pain for 2 months	Pancreatic body cancer with suspected invasion of the celiac trunk and superior mesenteric artery	305.9	AG + camrelizumab, FOLFIRINOX, GS + icaritin	18 months (SD then PD)
3	Female	77	Pancreatic mass found during a physical examination	Tail of the pancreas tumor	20.96	Laparoscopic surgery, AG + camrelizumab, icaritin	Alive (PR)
4	Male	74	Liver function abnormalities for 1 month were detected during a physical examination	Pancreatic head cancer with bile duct dilation	257.8	Lenvatinib + icaritin	Alive (slight tumor reduction)
5	Female	57	Left upper abdominal pain for 3 months	Pancreatic cancer invading the splenic artery and vein	49.66	Laparoscopic RAMPS surgery, AG + Camrelizumab, Lenvatinib + Icaritin	17 months (liver metastases resolved, then patient died)
6	Female	68	Rectal malignant lesion detected by colonoscopy for 2 weeks	Pancreatic caudal tumor with rectal metastasis	3000.9	GP regimen + icaritin, radiation therapy	Alive (SD)

PR, partial response; SD, stable disease; PD, progressive disease; OS, overall survival.

The use of lenvatinib in pancreatic cancer, although not extensively documented, has shown promise. One study reported success in treating pancreatic neuroendocrine tumors with lenvatinib, with an objective response rate of 44.2% and a disease control rate of 96.2%. However, this came with a challenging safety profile, as fatigue, hypertension, and diarrhea necessitated dose adjustments or discontinuation in 93.7% of patients [30]. In addition, lenvatinib has shown potential in inhibiting the growth of pancreatic cancer graft tumors, implying a link between microvessel density and the effectiveness of anti-angiogenic drugs [31]. As a multi-target inhibitor, lenvatinib blocks pathways including VEGFR2 (KDR)/VEGFR3 (Flt-4), FGFR1-4, PDGFRα/β, Kit (c-Kit), and RET (c-RET) and thus exhibits significant anti-tumor activity. Supplementary Table 2 provides an overview of patients’ treatment status following definitive diagnosis. Notably, many patients preferred lenvatinib over traditional chemotherapy or immunotherapy, citing its lower side-effect profile and broad spectrum of action. There has been a case of pancreatic cancer in which a certain degree of therapeutic effects was achieved using icaritin combined with lenvatinib in an elderly female patient, which displayed that the tumor lesion was significantly decreased by nearly 57.5% after treatment, and the extent of vascular involvement also diminished [25]. This case reinforces the idea that lenvatinib, when used in combination with icaritin, can potentially offer both therapeutic benefits and manageable side effects, improving patient tolerance to the regimen.

In the cases presented here, post-treatment imaging of all patients revealed varying but observable lesion reduction, supporting the preliminary efficacy of icaritin and lenvatinib or chemotherapy. This highlights the potential of combining these therapies as an effective strategy for treating advanced pancreatic cancer, particularly given their synergistic effects. IL-6 has been shown to play a crucial part in tumor growth by stimulating the release of angiogenic factors and promoting neovascularization [32]. Therefore, targeting IL-6 expression may impede tumor microangiogenesis, potentially boosting the efficacy of anti-angiogenic drugs.

The common adverse reactions of lenvatinib include hypertension, heart failure, and bleeding, and the side effects of AG or GS regimen are often gastrointestinal symptoms such as nausea and vomiting, as well as peripheral neuropathy and anemia. Throughout the entire treatment process, the side effects of icaritin combined with these drugs were tolerable. Most patients experience grade I-II leukopenia, thrombocytopenia, and anemia after using icaritin combined with chemotherapy or targeted therapy, which represented bone marrow suppression adverse reactions. Treatment of leukopenia and thrombocytopenia, as well as correction of anemia can alleviate these reactions. As a result, there were no cases of stopping treatment due to adverse reactions. This provides a valuable reference for future applications of icaritin as a supportive treatment in combination therapies. Although the above treatment benefited all the cases to varying degrees, there are several limitations in this study. First, the small sample size (only six cases) limits, which is difficult to explain the clear and effective role of icaritin in the treatment of pancreatic cancer. Additionally, this study lacks a control group, which makes it difficult to directly compare the efficacy of the combination treatment with standard therapies. This study carries an inherent risk of bias, as the lack of randomization introduces potential bias. Treatment decisions were influenced by factors such as prior treatments, patient preferences, and physician judgment, which could lead to bias in the results. Furthermore, all cases in this study were diagnosed based on clinical symptoms and imaging results, and some patients did not undergo immunohistochemical confirmation. This has potential limitations in the accurate diagnosis and differentiation of pancreatic cancer subtypes. Finally, there is a lack of sufficient evidence and clinical guidelines to support the combination therapy of icaritin with other drugs in the treatment of pancreatic cancer. Future research should focus on expanding the sample size and conducting multi-center randomized controlled trials to improve the reliability of the findings, and employing molecular diagnostics like immunohistochemistry and high-throughput sequencing will more accurately identify pancreatic cancer subtypes, guiding personalized treatment.

## 4. Conclusion

The combination of icaritin and lenvatinib or chemotherapy has shown preliminary potential in the case study, but due to the limited number of cases, it is difficult to confirm the universality of its therapeutic effect in treating advanced pancreatic cancer. On account of its favorable safety profile, characterized by minimal impact on liver and kidney function and a low risk of bone marrow suppression, icaritin as a supportive therapy makes it an optimal partner for use in combination therapies with targeted or immune drugs to treat advanced malignancies. These findings provide preliminary data to support future research on icaritin’s role in cancer treatment, especially for patients who are unable or reluctant to pursue first-line chemotherapy. Currently, there is an urgent need for large-sample, multi-center clinical trials or real-world studies to further validate the efficacy and tolerability of icaritin combined with chemotherapy or targeted therapies for advanced pancreatic cancer.

## Supplementary Material

Supplemental Material

## Data Availability

The data that support the findings of this study are available from the corresponding author, Defei Hong, upon reasonable request.
