# Supplementary material for: Icaritin and lenvatinib treatment for unresectable localized progressive pancreatic cancer: a report of six cases
Source: Ann Med. 2025 Jun 5;57(1):2512436. doi: 10.1080/07853890.2025.2512436 (PMC12143008; doi:10.1080/07853890.2025.2512436)
Supplement: Supplemental Material [file IANN_A_2512436_SM9400.zip › suppl_data/Supplementary Figure legends_0207.docx]

**Supplementary Figure caption**

**Supplementary Figure 1.** Abdominal imaging result after discontinuation of medication: increase of the active component and enlargement of the hooked area tumor (December 15, 2022).

**Supplementary Figure 2.** Imaging changes pre- and post-third chemotherapy (GS + Icaritin) from July 1, 2022, to February 9, 2023, and without liver metastasis.

**Supplementary Figure 3.** Enhanced abdominal CT imaging at admission: Cystic-solid mass in the pancreatic tail, suggesting malignancy, with potential metastasis(Red arrow points to tumors).

**Supplementary Figure 4.** Imaging Comparison Pre and Post AG and Camrelizumab Treatment: Hypodense hepatic masses in liver segments VII and VIII indicated by red arrows.

**Supplementary Figure 5.** Imaging results before and after icaritin treatment(Red arrow points to tumors). a+d). abdomen enhanced CT results before treatment showed a low-density mass in liver segments VII and VIII, measuring approximately 37*26 mm in liver VII, and multiple lymph nodes in the retroperitoneum, with the largest measuring about 11mm in short diameter. b+e). enhanced CT after approximately 2 months treatment of Iicaritin showed reduced low-density masses in liver segments VII and VIII and about 19*15 mm. c+f). Enhanced CT images taken approximately 3 months after icaritin treatment revealing a further reduction in size of the hepatic masses and about 16*10 mm in both liver segments VII and VIII.

**Supplementary Figure 6.** CT imaging of admission (2022.10.16): Multicystic soft tissue mass in the pancreatic head near the duodenal papilla, exhibiting slightly low enhancement and full pancreatic morphology with minimal exudation at the margins and dilated pancreatic duct(Red arrow points to tumors).

**Supplementary Figure 7.** Enhanced CT of admission (2021.11.04)(Red arrow points to tumors): Slightly hypodense mass in the caudal pancreas, with mild to moderate enhancement and poorly defined borders, approximately 3-3.5 cm in length; proximal segment of adjacent splenic artery encapsulated with rough edges and narrowed lumen and interrupted splenic vein with peri-gastric isthmic branching.

**Supplementary Figure 8.** Enhanced abdominal CT before and after postoperative chemotherapy of AG+Camrelizumab (Red arrow points to tumors). a,c) Post-pancrectomy with dense shadows in the surgical area and a soft tissue mass adjacent to the posterior peritoneal aorta. b,d) Absence of the pancreatic body tail and spleen post-surgery, with small linear shadows adjacent to the left posterior peritoneal aorta and normal morphology of the remaining pancreatic head. e) MR image showed tumor liver metastasis before the treatment of Lenvatinib+ Icaritin . f) Liver metastases almost disappeared after 1 month treatment.

**Supplementary Figure 9.** Abdominal Image. a)Abdominal MR image post-admission: malignant mass in caudal of the pancreas, involving adjacent intestinal ducts; slight thickening of the right diaphragm and overlying membrane; ascites; and swollen lymph nodes in the intra-abdominal cavity and right cardiac septal angle. b)Enhanced CT image post-additional Icaritin treatment over 6 months: 1. Chest: Reduced lymph node size in the right cardiac diaphragmatic angle. 2. Abdomen: Slight reduction in pancreatic tail lesion; decreased peritoneal thickening with fewer nodules; resorption of abdominal ascites; reduced intra-abdominal exudate; liver and kidney cysts unchanged. 3. Pelvis: Pelvic fluid absorption with no new abnormalities.
