# Supplementary material for: Icaritin and lenvatinib treatment for unresectable localized progressive pancreatic cancer: a report of six cases
Source: Ann Med. 2025 Jun 5;57(1):2512436. doi: 10.1080/07853890.2025.2512436 (PMC12143008; doi:10.1080/07853890.2025.2512436)
Supplement: Supplemental Material [file IANN_A_2512436_SM9400.zip › suppl_data/Supplementary_Table_1.docx]

**Supplementary Table 1.** The imaging review shows the progressive shrinkage of liver metastases and retroperitoneal lymph nodes of case 3 after taking Icaritin.

| Imaging site | Timing | | |
| --- | --- | --- | --- |
|  | 2023.02.13 | 2023.04.09 | 2023.05.24 |
| Liver metastases (mm) | 37*26 | 19*15 | 16*10 |
| Retroperitoneal lymph nodes (mm) | 11 | 9 | 8 |
