# Supplementary material for: Icaritin and lenvatinib treatment for unresectable localized progressive pancreatic cancer: a report of six cases
Source: Ann Med. 2025 Jun 5;57(1):2512436. doi: 10.1080/07853890.2025.2512436 (PMC12143008; doi:10.1080/07853890.2025.2512436)
Supplement: Supplemental Material [file IANN_A_2512436_SM9400.zip › suppl_data/Supplementary_Table_2.docx]

**Supplementary Table 2.** Treatment status after the definite diagnosis of the patients.

| **Case** | **Date** | **Treatment** | **CA19-9(IU/mL)** | **Imaging** | **Tumor Staging** | **Post-Treatment Evaluation** |
| --- | --- | --- | --- | --- | --- | --- |
| 1 | 2022.08.15 | Lenvatinib + Icaritin*2 months | 69.80 | 57*31mm | T3N0M0, Stage IIA | PR |
|  | 2022.09.24 |  | 30.52 | 35*28mm |  | PR |
|  | 2022.10.14 |  | Unchecked | 31*25mm | T2N0M0, Stage IB | Continuous PR |
|  | Abdominal CT re-examination at external hospital 2022.12.22 | Discontinued medication | 501.60 | An increase of the active ingredient and an enlargement of tumors in the uncinate process of the pancreas |  | PR compared to initial treatment and PD compared to discharging from our hospital |
| 2 | 2021.09.01  2021.09.01-2021.12.01 | Baseline  AG +Camrelizumab*4 cycles | 305.90  2021.09.10/161;2021.09.21/155.9;2021.10.29/65.01;2021.1.24/33.06;2021.12.01/21.83 | 27*28mm  20*14mm(2021.12.10) | T4NxM1, Stage IV | Imaging on 2021.12.1 shows a 28.6% reduction in lesion size, clinical SD |
|  | 2021.12.01-2022.07.01 | FOLFIRINOX*6cycles | 2021.12.22/23.76;2022.01.19/20.60;2022.02.19/21.86;2022.03.18/34.06;2022.04.15/20.70;2022.05.23/37.35;2022.07.01/123.50 | 20*14mm(2021.12.10)  2022.07.01 3cm of the pancreatic body |  | Imaging on 2022.7.1 shows a 50% increase in lesion size, clinical PD |
|  | 2022.07.01-2022.12.12-2023.02.09 | GS+Icaritin | 2022.07.30/296.80;2022.08.23/252.20;2022.09.17/188.10;2022.11.04/305.80;2022.11.26/384.50;2023.02.17/979 | 2022.12.12 3cm of the pancreatic body;2023.02.09 4.5cm of the pancreatic body |  | Imaging after 7 months shows SD |
|  | 2023.03.10 |  |  |  |  | Death |
| 3 | 2022.05.16 | Laparoscopic radical pancreatectomy + splenectomy + partial colectomy + adhesiolysis | 20.96 | 64*41mm | pT3N0Mx, Stage II |  |
|  | 2022.06.18 | AG*1cycles | 5.25 | The changes after pancreatectomy without recurrence or metastasis(2022.06.19CT) |  | Refusing chemotherapy, followed by recurrence with intrahepatic metastasis |
|  | 2023.02.13 2.17 formal medication,2023.05.24 last medication | AG+Camrelizumab*6cycles | 2023.2.12/38.48;2023.2.17/45.60;2023.3.14/18.78;2023.4.9/8.20;2023.5.10/6.66 | 2023.02.13 37*26mm;2023.04.09 19*15mm;2023.05.25 16*10mm | Recurrence, Stage IV | PR |
|  | 2022.08.08-2022.10.08 | Icaritin | 2022.6.25/5.25;2022.7.23/6.67 | 08.09 PET_CT; low-density nodules such as liver, considering tumor activity inhibition (unmeasured size);11.23 MR:7mm nodule in liver segment VII, considering metastasis, smaller than 5.25 |  | Imaging after 2 months shows a reduction in liver metastasis and retroperitoneal lymph nodes. Last follow-up on 2023.07.25, clinical continuous PR |
|  | 2023.12.04 survival |  |  |  |  |  |
| 4 | 2022.10.18 | baseline | 257.80 | 2022.10.16 34*31mm | T4N0M0, Stage III | Suggest surgical patients to refuse and refuse all chemotherapy |
|  | 2022.10.24-2023.05.08 | Lenvatinib+Icaritin | 2022.11.25/335;2023.01.03/350.10;2023.01.30/399.60;2023.03.02/543;2023.05.02/619 | 31*28mm 2022.11.25 |  | 1-month post-treatment imaging shows a slight reduction in tumor lesions; no liver or kidney damage during treatment; efficacy evaluation SD |
|  | 2023.01.03 |  | 350.10 | 30*25mm2023.01.04 |  | Efficacy evaluation SD |
|  | 2023.03.02 |  | 543 | 40*35mm 2023.03.03 |  | Efficacy evaluation PD |
|  | 2023.05.02 |  | 619 | 40*35mm 2023.05.03 |  | Efficacy evaluation SD |
|  | 2023.06.11 |  | 779.40 | 40*35mm 2023.06.11 |  | Efficacy evaluation SD |
|  | 2023.09.05Death |  |  |  |  |  |
| 5 | 2021.11.06 | Operation 2021.11.11 | 49.66 |  | pT2N1Mx, Stage II B |  |
|  | 2022.01.26-2022.08.15 | AG+Camrelizumab | 2022.01.19/113.80;2022.03.12/89.12;2022.04.12/44.12;2022.04.28/42.02;2022.05.06/28.05;2022.05.28/30.23;2022.07.04/27.50;2022.08.13/50.01 | 2022.01.19 CT: After the operation of pancreatic cancer, retroperitoneal masses surrounded the abdominal aorta and left renal artery;2022.03.15 CT: similar to the former condition on; 2022.05.31 CT: similar to the former condition;2022.08.15 CT: similar to the former condition |  | Imaging shows tumor progression(after the first follow-up, chemotherapy combined with immunity, long-term SD) |
|  | 2022.10.28 | Lenvatinib+Icaritin | 2022.10.27/238.50;2022.11.29/353.00 | 2022.10.19 abdominal CT: after the operation of pancreatic cancer, multiple metastases occurred in the mesangium, omentum, peritoneum, and abdominal wall, with obvious progress compared with 8.15;2022.11.29 after the operation of pancreatic cancer, the metastasis of liver margin, peritoneum and abdominal wall is weakened compared with the previous enhancement(the liver metastasis is relieved, and the activity of abdominal wall and peritoneal metastasis is significantly weakened) |  | Imaging examination confirmed the recurrence of the disease, and liver metastasis had already existed before second-line treatment. After treatment, a follow-up examination showed that the liver metastasis had improved |
|  | 2022.12.31 |  | 2023.02.03/2049.00;2023.03.17/4614.00 | 2023.01.01 abdominal CT: pancreatic cancer had multiple metastases after surgery, significantly more advanced than 2022.11.29;23.02.04 abdominal CT: pancreatic cancer had multiple metastases after operation, which is partly larger than 01.01, and partly weaker than 01.01 |  | 2022.12.31 CT Imaging: rapid tumor progression without subsequent treatment |
|  | 2023.04.16 |  |  |  |  | Death |
| 6 | 2022.08.08-2022.08.26 | Baseline-pathological diagnosis, imaging staging confirmation | 3000.90 | 29×25×43mm at the tail of pancreatic,2022-08-19;30×35mm 4mm from anal edge till rectum | IV AJCCth | The tail of the pancreatic mass, at the dismal of the transverse colon and rectum, appeared middle differentiation, adenocarcinoma metastasis |
|  | 2022.09.10 | GP | 2025.0, 2022-09-17;288.0 2022-11-29 | Lung metastasis, no the size of the tumor, PET-CT(2022-11-22) |  | PD |
|  | 2022.12.04 | GP+Icaritin | 181.01, 2022-12-29 | No the size of the tumor(2023.03.16) |  | PD-multiple masses in the right lung and right oblique fissure; The lesion in the lower segment of the rectum near the anus is larger than before, with multiple abnormal signal shadows in the rectum, and increased glucose metabolism, indicating the progression of the disease |
|  | 2023.04.XX- | GP+Icaritin +radiotherapy(50Gy/25f) | 92.3, 2022-04-19 | No the size of the tumor(2023-07-06) |  | PD-PET-CT the lesion range at the tail of the pancreatic is slightly smaller than before, the lesion at the intestinal wall is significantly smaller, the glucose metabolism is still high, the range of abnormal signal shadows in the right lung is larger than before |
|  | 2023.07.19 | FOLFIRINOX+Icaritin | 5.86 | No the size of the tumor(2023-05-24) |  | The lesion in the pancreatic and transverse colon is smaller than before |

PR: Partial Response; SD: Stable Disease; PD: Progressive Disease; OS: Overall Survival
