# Supplementary figures and images for: Icaritin and lenvatinib treatment for unresectable localized progressive pancreatic cancer: a report of six cases
Source: Ann Med. 2025 Jun 5;57(1):2512436. doi: 10.1080/07853890.2025.2512436 (PMC12143008; doi:10.1080/07853890.2025.2512436)

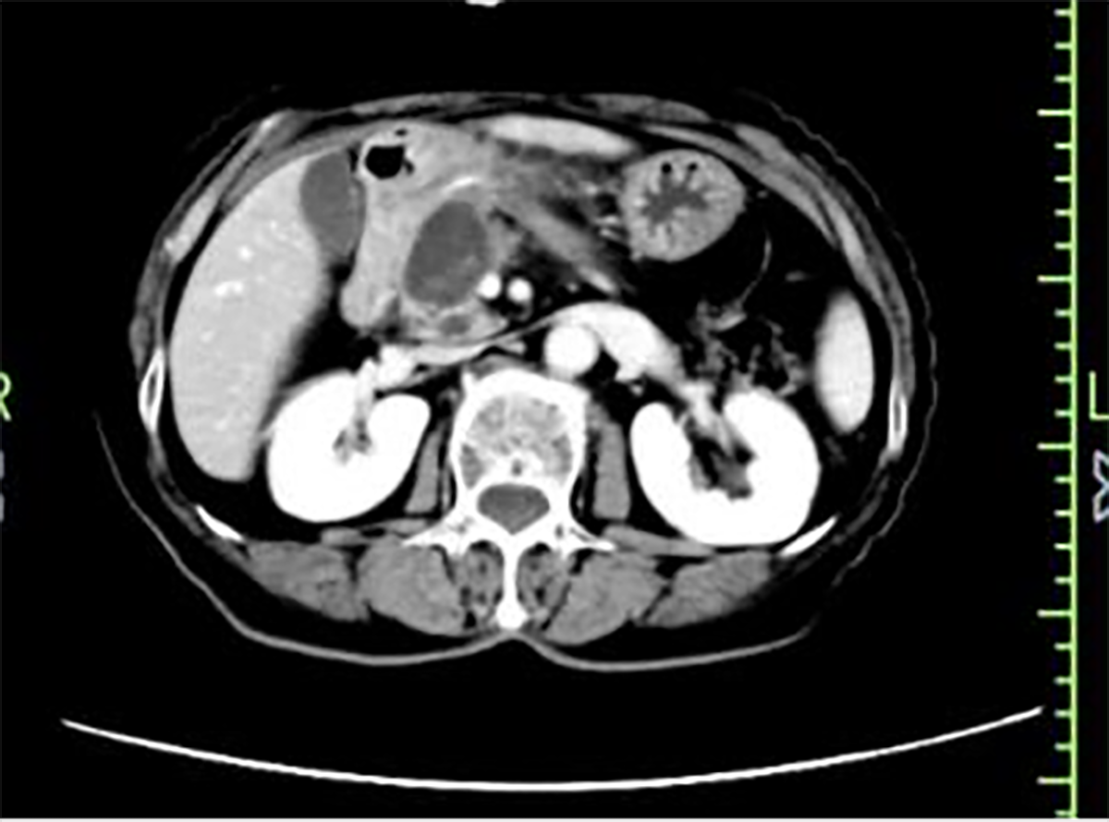

Supplement: Supplemental Material [file IANN_A_2512436_SM9400.zip › suppl_data/Supplementary Figure 1.tif]

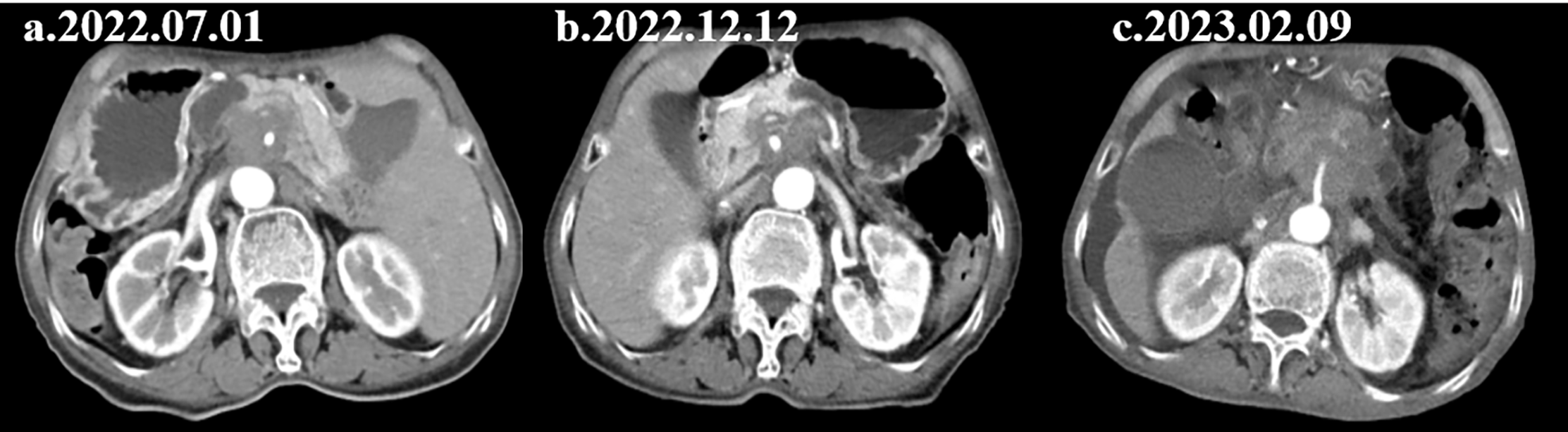

Supplement: Supplemental Material [file IANN_A_2512436_SM9400.zip › suppl_data/Supplementary Figure 2.tif]

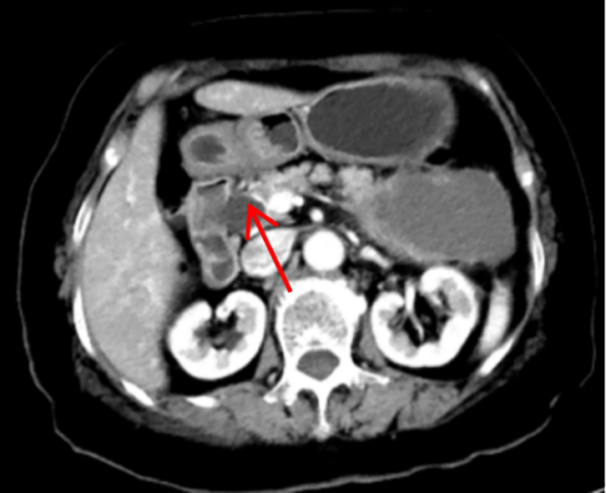

Supplement: Supplemental Material [file IANN_A_2512436_SM9400.zip › suppl_data/Supplementary Figure 3.tif]

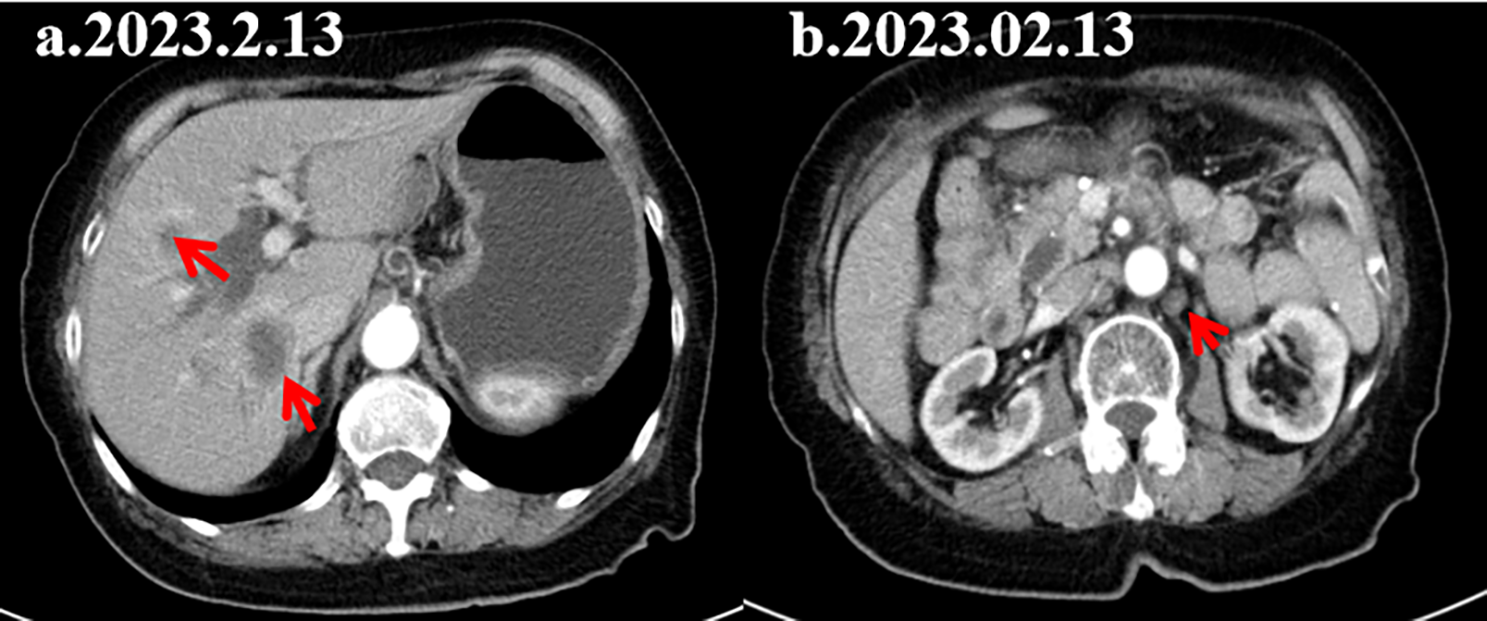

Supplement: Supplemental Material [file IANN_A_2512436_SM9400.zip › suppl_data/Supplementary Figure 4.tif]

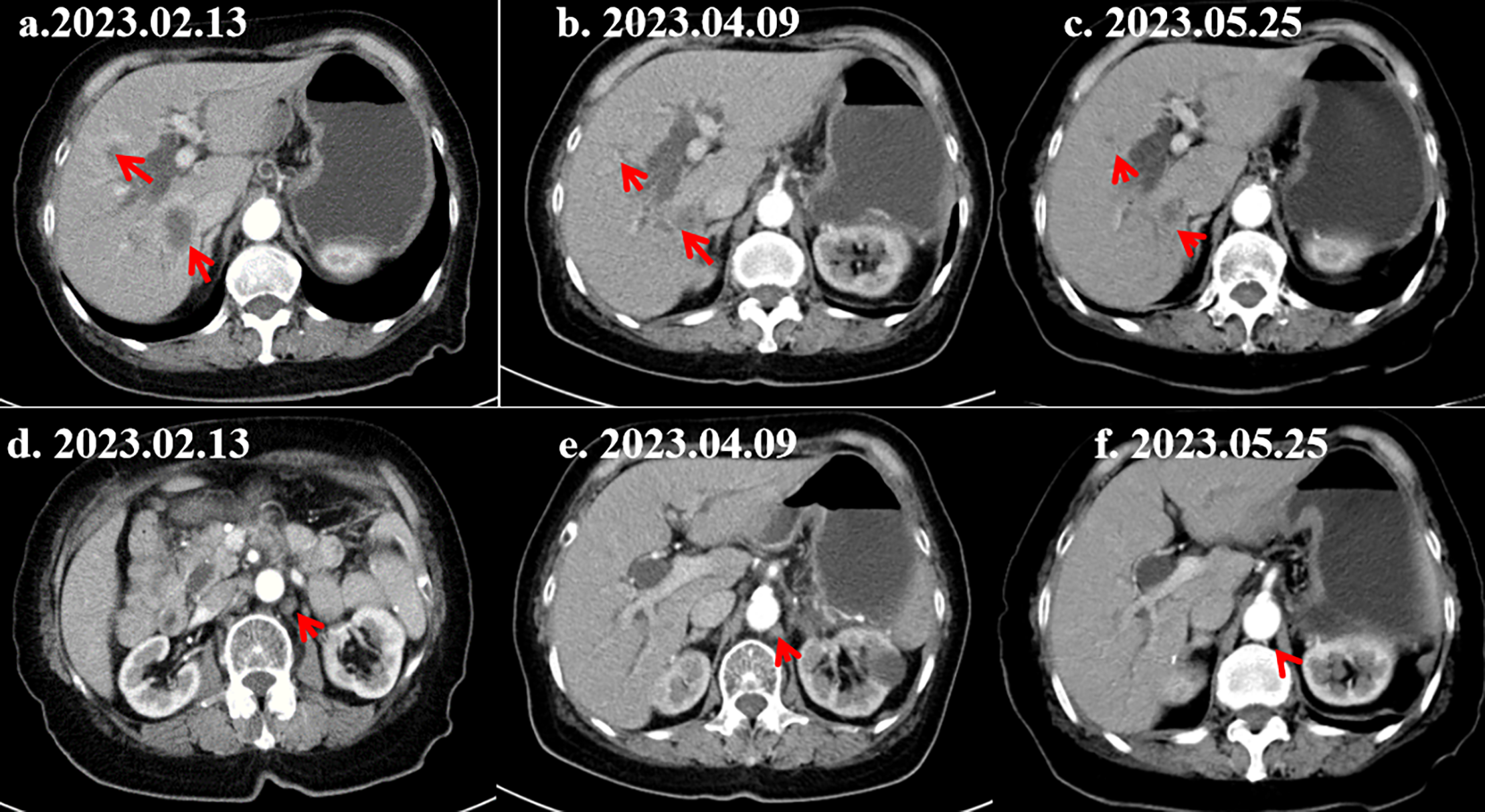

Supplement: Supplemental Material [file IANN_A_2512436_SM9400.zip › suppl_data/Supplementary Figure 5.tif]

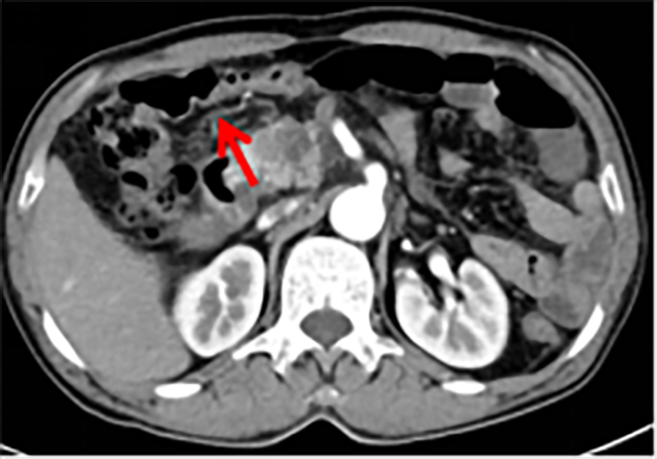

Supplement: Supplemental Material [file IANN_A_2512436_SM9400.zip › suppl_data/Supplementary Figure 6.tif]

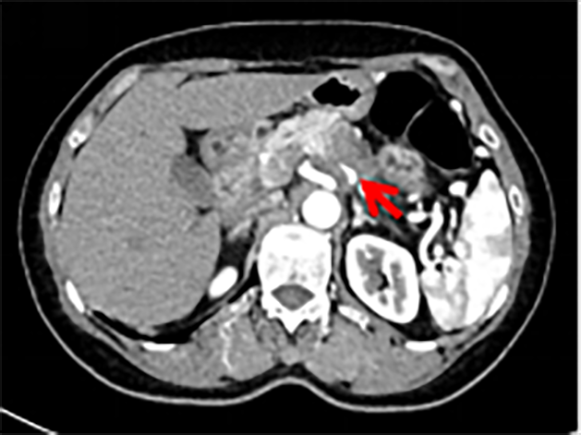

Supplement: Supplemental Material [file IANN_A_2512436_SM9400.zip › suppl_data/Supplementary Figure 7.tif]

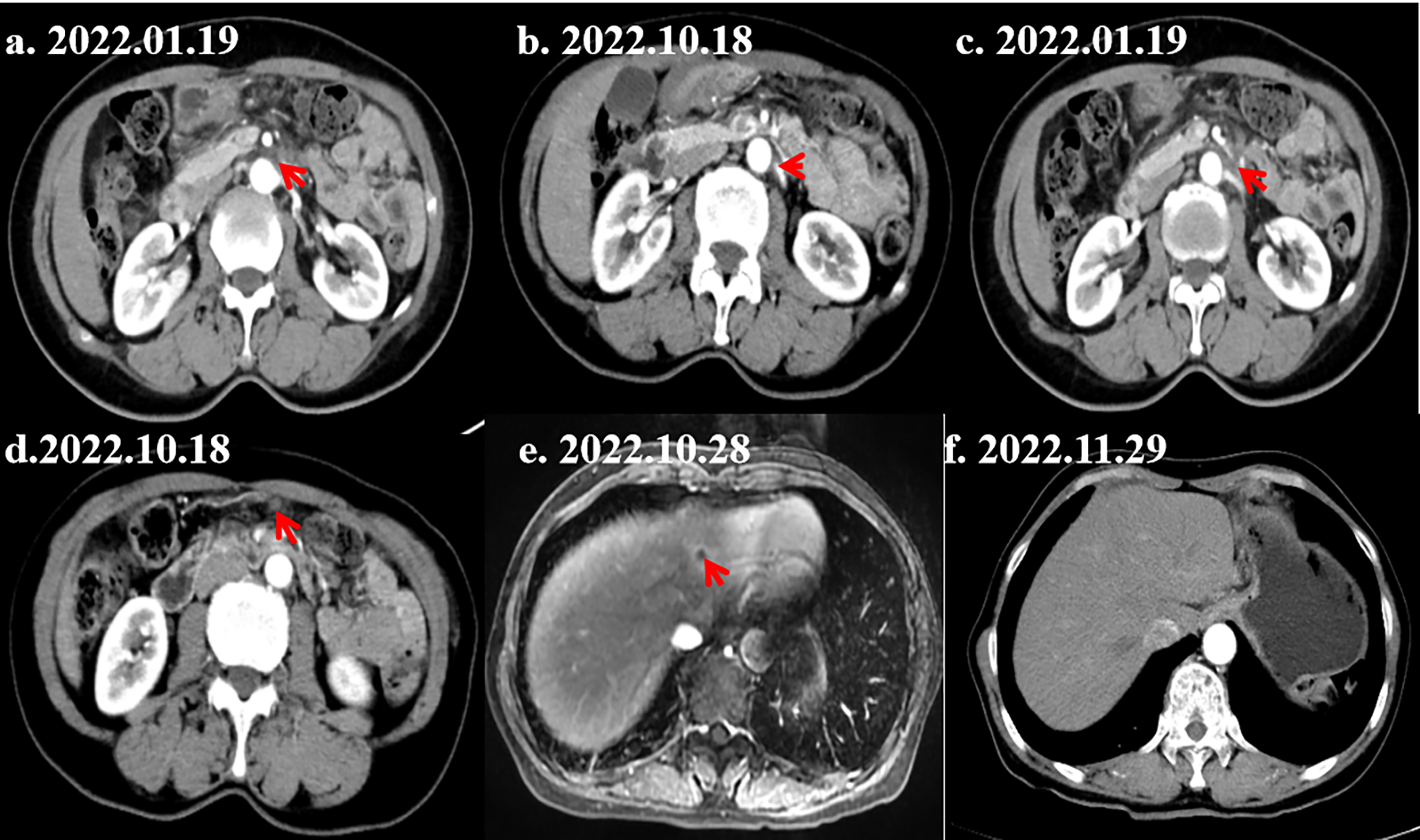

Supplement: Supplemental Material [file IANN_A_2512436_SM9400.zip › suppl_data/Supplementary Figure 8.tif]

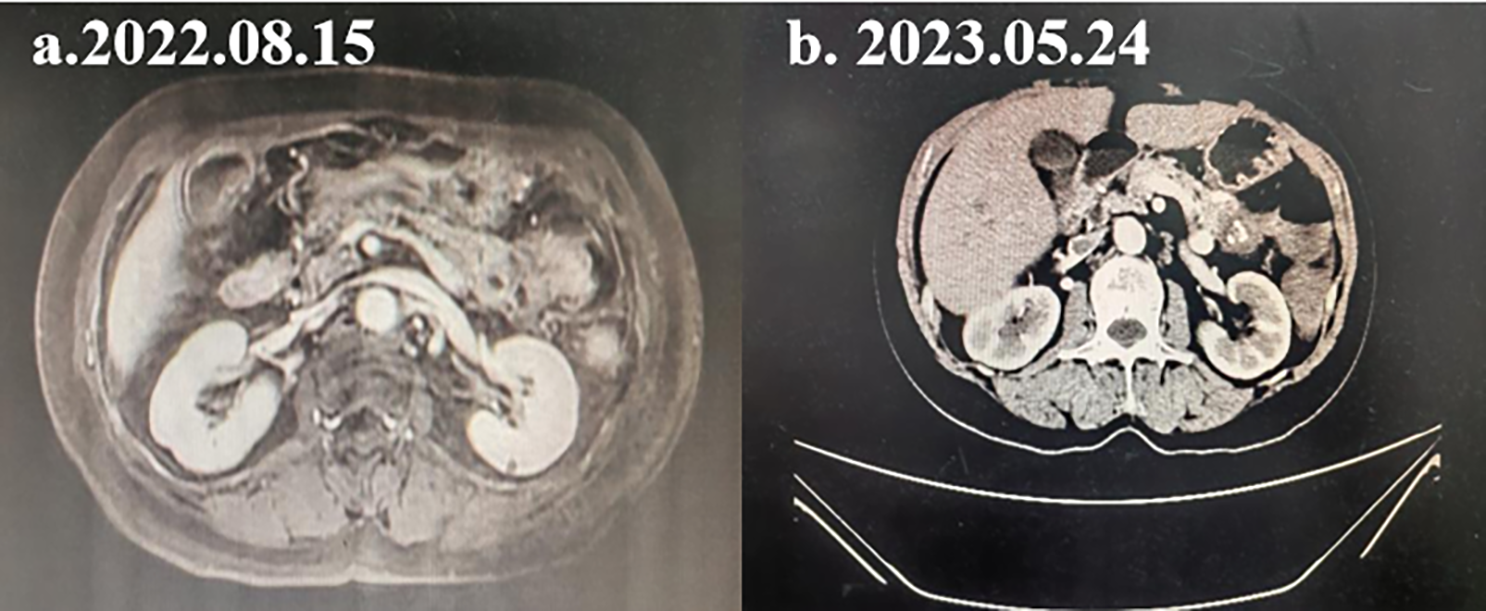

Supplement: Supplemental Material [file IANN_A_2512436_SM9400.zip › suppl_data/Supplementary Figure 9.tif]
